# Safety and Efficacy of Digital Check-in and Triage Kiosks in Emergency Departments: Systematic Review

**DOI:** 10.2196/69528

**Published:** 2025-05-21

**Authors:** Elena Lammila-Escalera, Geva Greenfield, Reham Aldakhil, Hei Ming Mak, Himani Sehgal, Ana Luisa Neves, Mark J Harmon, Azeem Majeed, Benedict Hayhoe

**Affiliations:** 1 Global Digital Health Unit Department for Primary Care and Public Health Imperial College London London United Kingdom; 2 School of Medicine University of Liverpool Liverpool United Kingdom; 3 Lewisham and Greenwich NHS Trust London United Kingdom; 4 Department for Primary Care and Public Health Imperial College London London United Kingdom

**Keywords:** emergency services, hospital, triage, self-service kiosk, urgent care

## Abstract

**Background:**

Emergency departments (EDs) globally face unprecedented pressures due to aging populations, multimorbidity, and staff shortages. In response, health systems are adopting technological solutions such as digital kiosks to reduce wait times, improve patient flow, and alleviate overcrowding. These tools can automate patient check-in and assist with triage, helping to reduce variability in assessments and identify individuals with urgent needs sooner. However, it remains unclear whether the potential time-saving benefits of these innovations translate into improved patient outcomes and safety.

**Objective:**

This systematic review aims to summarize the safety and efficacy impacts of digital check-in and triage kiosks compared with traditional nurse-led triage methods in EDs.

**Methods:**

Comprehensive searches were conducted in MEDLINE, EMBASE, and Web of Science. A narrative synthesis was carried out to evaluate the impact on patient safety (eg, agreement rate, accuracy, sensitivity, and specificity) and efficacy (eg, operational efficiency and patient flow). The quality of the studies was assessed using the National Heart, Lung, and Blood Institute quality assessment tools.

**Results:**

A total of 5 studies, comprising 47,778 patients and 310,249 ED visits, were included. Out of these 5 studies, 3 focused on self-check-in kiosks, one on self-triage kiosks, and another on technology combining both. Among 5 studies, 2 evaluated safety, reporting high sensitivity for predicting high-acuity outcomes (up to 88.5%) and low under-triage rates (8.0%-10.1%) but poor agreement with nurse-assigned triage scores (27.0%-30.7%). Specificity for low-acuity cases was variable, with one study reporting as low as 27.2% accuracy. Of the 5 studies, 4 examined efficacy, reporting high over-triage rates (59.2%-65.0%) and mixed impacts on waiting times. While 2 studies found significant reductions in time-to-physician and time-to-triage, others reported no significant improvements following adjustments. Kiosks demonstrated high usability, with one study reporting 97% uptake among ED attendees.

**Conclusions:**

Evidence on the safety and efficacy of digital check-in and triage kiosks remains sparse. Based on the limited number of studies available, digital kiosks appear effective in accurately identifying high-acuity patients; however, their impact on operational efficiency measures is unclear. High over-triage rates and poor concordance with nurse-assigned triage scores may limit their practical application in busy ED settings. Further research is required to evaluate long-term outcomes, implementation across diverse health care contexts, and integration into ED workflows to better understand how digital kiosks can safely and effectively help address the growing demand for EDs.

**Trial Registration:**

PROSPERO CRD42024481506; https://www.crd.york.ac.uk/PROSPERO/view/CRD42024481506

**International Registered Report Identifier (IRRID):**

RR2-10.1136/bmjopen-2024-084506

## Introduction

Emergency departments (EDs) worldwide are experiencing unprecedented demand. In the United Kingdom, urgent hospital admissions have increased by 42% over the past decade, leading to long wait times and ED overcrowding [1-4]. Excessive demand and overcrowding in EDs are a significant barrier to the timeliness of care and are detrimental to staff well-being, ultimately compromising patient safety [2,5,6].

An aging population, increased frailty, and a rising number of individuals with multiple long-term conditions drive this international crisis [7-10]. These pressures restrict access to other health care services, making patients rely more on EDs for nonurgent and primary care-sensitive visits [11,12]. In addition, the strain on EDs leads to higher hospital readmission rates, as many patients return due to the acute nature of initial treatment or lack of follow-up care arrangements [13]. In response, health systems globally are exploring technological innovations to address this demand management issue.

One innovation in EDs is the introduction of digital check-in kiosks, which automate the initial check-in process [14]. These enable patients to enter information such as demographic details, presenting conditions, and medical history immediately upon arrival [15,16]. Integrating kiosks, electronic health records, or online patient portals streamlines operations by eliminating redundant administrative tasks and reducing the workload of clinical staff, enabling them to prioritize and deliver appropriate care [15,17].

In response to the COVID-19 pandemic, health care systems rapidly deployed electronic self-triage tools, highlighting their usability [18,19]. When validated by a triage nurse or other clinician, some self-service kiosks suggest priority scores based on patient-entered clinical details to help reduce variability in triage assessments and identify individuals with more urgent clinical problems sooner [20]. This function may also support the ability to stream patients to the most appropriate clinical pathway within EDs (eg, same-day emergency care) or alternative units or clinical areas (eg, urgent care centers or minor injury units). However, misclassification remains a significant concern, prompting questions about whether the time-saving benefits of digital triage extend to improvements in patient outcomes and safety. Studies have reported instances of under-triage and over-triage with digital triage systems [21]. Under-triage is perhaps the more safety-critical concern, potentially delaying care for individuals with urgent clinical problems. Still, over-triage may unnecessarily escalate low-acuity cases and contribute to reduced overall efficiency, with consequential impact on individuals in greater immediate need [20].

Previous research has broadly explored kiosks, examining their roles in prevention, counseling, and telemedicine [22-24]. However, systematic reviews have yet to provide a granular focus on self-service check-in kiosks in EDs or urgent care settings. While past reviews have assessed digital triage accuracy, they did not use kiosks as a delivery method [20,25,26]. Although current evidence indicates no harm to patient safety, further research is required to evaluate how accurately these systems assess patient acuity and their long-term impact [25,26].

Consequently, this systematic review aims to summarize the evidence on the safety and efficacy of self-service kiosks used for self-check-in and self-triage in EDs. As kiosk adoption becomes more common, it is crucial to understand its broader impact and inform the widespread integration of these tools and best practices.

## Methods

### Overview

This systematic review followed the PRISMA (Preferred Reporting Items for Systematic Reviews and Meta-Analysis) guidelines (Multimedia Appendix 1) [27]. A protocol was registered in PROSPERO (International Prospective Register of Systematic Reviews; registration number CRD42024481506) and was recently published [28].

### Eligibility Criteria

The criteria for inclusion and exclusion were structured according to the Population, Intervention, Comparator, Outcome, and Study Designs (PICOS) framework (Table 1). This systematic review aimed to evaluate the safety and efficacy of self-service kiosks in EDs compared with traditional nurse-led triage methods, focusing on studies from 2004 to 2024. As self-service kiosks were first introduced in the mid-2000s, this timeframe was chosen to ensure all relevant evidence was captured [22].

**Table 1:** Inclusion criteria for eligibility.

PICOS^a^ Framework	Inclusion criteria
Population	Adult patients and health care staff within ED^b^ setting.
Intervention	Self-service kiosks that act as either a digital triage, check-in tool (eg, a kiosk with a touch-screen or other digital input method that allows patients to: enter demographic and clinical information, undergo initial symptom assessment, be categorized into urgency or risk categories, check in to the ED or alert staff to their arrival), or both.
Comparator	Traditional methods for check-in or triage (using triage nurses) without the addition of a self-service kiosk.
Outcomes	Quantitative measures assessing safety (eg, accuracy of triage and adverse events) and assessing efficacy (eg, workflow, staff workload, triage time, and overall operational efficiency).
Study design	Quantitative studies and mixed-method studies with a significant quantitative component, including RCTs^c^, case-control studies, cohort studies, and cross-sectional studies.

^a^PICOS: Population, Intervention, Comparator, Outcome, Study design.

^b^ED: emergency department.

^b^RCT: randomized controlled trial.

### Information Sources and Search Strategy

MEDLINE (Ovid), Web of Science, and EMBASE (Ovid) were searched to ensure that all biomedical, digital health, and public health literature was captured. The search strategy, created alongside an expert Librarian, incorporated a variety of keywords, Boolean operators, and Medical Subject Headings (MeSH) terms and was customized to each database (Multimedia Appendix 2).

### Selection and Data Collection Process

Two independent reviewers deduplicated and screened studies identified through database searches using the screening software Covidence. Any discrepancies were resolved through discussion or with the input of a third researcher.

### Data Extraction Process

Two independent researchers (HMM and RA) extracted all relevant data (eg, study identifier, study design and duration, self-service kiosk description, comparator, and outcomes) and compiled them into a standardized table.

### Risk of Bias Assessment

HMM assessed the risk of bias in included studies using the National Heart, Lung, and Blood Institute (NHLBI) Quality Assessment Tool for observational cohort and cross-sectional studies [29]. RA then verified the risk of bias assessment.

### Synthesis Methods

Due to the heterogeneity in study designs, a meta-analysis was deemed impractical. A narrative synthesis was used to gather data on the clinical safety and efficacy of implementing self-service kiosks in EDs.

### Data Items and Effect Measures

Outcomes under examination include those related to patient safety and efficacy (Table 2). For this review, under-triage is categorized within the safety domain, whereas over-triage is classified within the efficacy domain. Other outcomes will be collated as reported.

**Table 2:** Outcomes under investigation.

Domain and outcomes		Description
**Safety**		
	Agreement	Agreement rate (percentage or kappa statistic) between kiosk-generated and nurse-assigned triage scores.
	Sensitivity	The proportion or percentage of true urgent cases correctly identified by the kiosk, in comparison to the nurse-assigned scores.
	Under triage	Percentage of cases where the kiosk assigned a lower urgency level than the nurse.
	Adverse events	Complications or harm resulting from delayed care, triage, or incorrect triage. These could be measured using patient surveys or by reviewing patient records for complications, delays in care, or outcomes such as readmissions.
**Efficacy**		
	Workflow management	Assessing how the kiosks impacted patient flow, administrative tasks, and extent of over-triage (incorrectly identifying low-risk cases as high-risk). This could be quantified by time motion studies or by staff surveys.
	Staff workload	Measuring changes in staff involvement by time tracking or by workload assessment surveys.
	Over-triage	Percentage of cases where the kiosk assigned a higher urgency level to the patient than the nurse.
	Time to physician	The time from ED^a^ arrival (including check-in and triage) to the patient being seen by a clinician. This could be measured by direct observation or by reviewing time logs or stamps.
	Time-to-triage	The time taken from ED arrival to the completion of triage. This could be measured by direct observation or by reviewing time logs or stamps.
	Time-to-first identification	The time taken from ED arrival to kiosk completion or the interval from arrival to triage. This could be measured by direct observation or by reviewing time logs or stamps.
	Usability	The percentage of patients that used the kiosk.

^a^ED: emergency department.

## Results

### Overview

**Figure 1:**
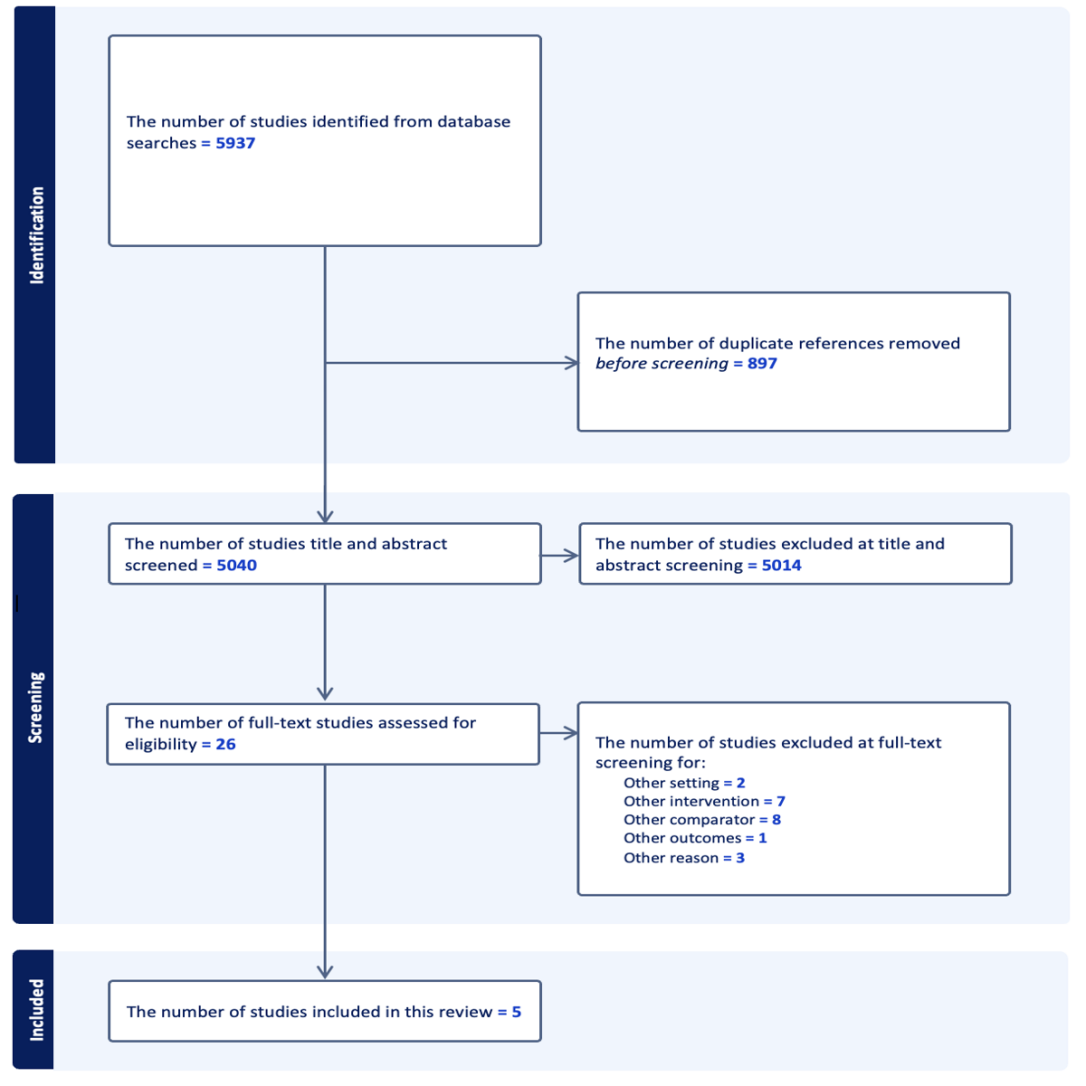
PRISMA flow diagram of included studies.

Searches identified 5937 eligible studies (Figure 1). Of the 26 studies selected for full-text screening, 21 were excluded due to the comparator, intervention, setting, and outcomes. Five articles were, therefore, eligible for inclusion in this review [30-34].

### Study Characteristics

The 5 studies included (Table 3) were conducted between 2019 and 2022 across Canada [30,34], the United States [32,33], and the United Kingdom [31] and included 47,778 patients and 310,249 ED visits. Study durations ranged from 10 weeks to 2 years. The studies comprised various designs: 1 cross-sectional [32], 1 pilot study [34], 2 retrospective analyses [31,33], and 1 randomized controlled trial [30]. All studies included patients of diverse ages, genders, comorbidities, and socioeconomic demographics.

**Table 3:** Study characteristics.

Study ID (Author, year)	Study period	Country	Study design	Sample size	Setting	Study hours	Population	Exclusions	Comparator
Coyle et al, 2019 [30]	10 weeks	Canada	Randomized controlled trial, prospective	3561 patients	ED^a^ of a tertiary care academic hospital treating an average	10:30 AM to 6:30 PM on weekdays	Patients attending ED	Patients who arrived via ambulance or who were CTAS^b^ Level 1	Routine nurse-initiated patient identification
Dickson et al, 2022 [31]	7 months	United Kingdom	Retrospective, observational	43,788 patients	2 hospitals with a combined annual ED attendance of over 135,000	Not reported	Patients aged >18 years, attending ED	Patients attending ED by ambulance or those aged <18 years	Nurse-led triage using the MTS^c^
Mahmood et al, 2020 [32]	1 year	United States	Cross-sectional, retrospective, observational	40,528 ED visits	480 EDs (outpatient departments and ambulatory surgical centers, short-stay and general hospitals, and freestanding ambulatory surgical centers	Not applicable	480 EDs participating in the National Ambulatory Medical Care Survey	Federally owned hospitals, including Veterans Affairs and military hospitals	Routine nurse-initiated patient identification
Alishahi Tabriz et al, 2020 [33]	2 years	United States	Retrospective analysis, observational	269,721 ED visits	EDs from 2826 hospitals (outpatient departments and ambulatory surgical centers, short-stay and general hospitals, and freestanding ambulatory surgical centers)	Not applicable	EDs participating in the National Ambulatory Medical Care Survey (2007-2015)	Federally owned hospitals, including Veterans Affairs and military hospitals	Routine nurse-initiated patient identification
Trivedi et al, 2021 [34]	Not applicable	Canada	Pilot, prospective, observational	429 patients	ED of a tertiary care center serving a population of 400,000	Weekdays, weekends, and into the evening on occasion. Not after midnight	Patients aged >16 years, attending ED	Patients bought by ambulance, those not speaking or reading English, and those aged <16 years	Nurse-led triage using the CTAS

^a^ED: emergency department.

^b^CTAS: Canadian Triage and Acuity Scale.

^c^MTS: Manchester Triage Score.

### Risk of Bias

A total of 4 studies were rated fair quality [31-34] while one was poor [30] (Multimedia Appendix 3 [30-35]). The lower rating was attributed to participant recruitment, sample size justification, and study timeframe. This may impact selection bias and generalizability, with inadequate sample size justification affecting statistical power and the absence of a specified timeframe introducing uncertainty about long-term effects. All cross-sectional and cohort studies clearly defined their objectives and outcomes, measuring the exposure of interest before the outcome [31-34]. However, repeat exposure assessments, reporting of unspecified outcomes, sample size justification, and participant blinding were less reliably reported.

### Description of Self-Service Kiosk

All studies focused on self-service kiosks (Table 4). Out of 5 studies, 3 assessed kiosks with a digital check-in feature [30,32,33], while another evaluated a digital triage function [34]. 1 study examined kiosks integrating both digital check-in and triage components [31]. Digital triage was conducted using algorithmic questionnaires or branched decision logic.

**Table 4:** Description of the self-service kiosk by function.

Digital kiosk and study ID		Description
**Self-check-in kiosk**		
	Coyle et al, 2015 [30]	Kiosk check-in, with either a prepopulated “chief complaint” or free-text box. Information sent to nurses for triage.
	Mahmood et al, 2020 [32]	Patients could check into ED^a^ without the help of medical administrators, using kiosk self-check-in.
	Tabriz et al, 2020 [33]	Patients could check into ED without the help of medical administrators, using kiosk self-check-in.
**Self-triage kiosk**		
	Trivedi et al, 2021 [34]	Electronic tablet AGST^b^ score. The algorithmic questionnaire (comprised of Yes/No questions) was completed by the patient or caregiver on the tablet by touching the screen to choose the answer most appropriate to their situation. Subjects were asked to predict whether they would require admission to the hospital. At the end of the questionnaire, the AGST score was assigned.
**Combined self-check-in and self-triage kiosk**		
	Dickson et al, 2022 [31]	eTriage is an automated digital check-in and triage solution. Patients who present to the ED provide their demographic details and then select a reason for attendance. The software is based on branched algorithmic decision logic with key discriminators and flagging which prompts further questions regarding the patient’s attendance. The platform mirrors the MTS^c^ in that it allocates the patient into one of five suggested priority groups.

^a^ED: emergency department.

^b^AGST: algorithm-generated self-triage.

^c^MTS: Manchester Triage Score.

### Reported Outcomes

#### Safety

2 of the 5 included studies evaluated safety outcomes for self-triage and combined technology-enabled kiosks [31,34]. Both studies highlighted high sensitivity for predicting high-acuity outcomes, compared with nurse-led triage [34]. For example, Dickson et al [31] reported 88.5% sensitivity for the combined kiosk, compared with 53.8% for the MTS assigned by nurses.

In addition, one study reported that these kiosks demonstrated high specificity for low-acuity outcomes, achieving 88.5% compared with 80.6% for nurse-assigned scores [31]. However, Trivedi et al [34] found that the self-triage kiosk correctly identified only 27.2% of low-acuity cases. Both studies observed low undertriage rates, with 8.0% reported by Trivedi et al [34] and 10.1% by Dickson et al [31]. Agreement between kiosk-generated and nurse-assigned triage scores was poor, with 27.0% and 30.7% concordance rates, respectively.

#### Efficacy

A total of 4 studies reported efficacy outcomes for self-check-in, self-triage, and combined kiosk interventions [30-32,34]. Coyle et al [30] reported a high usability rate, with 97% of ED attendees using the kiosk. Of the 4 studies, 2 also observed shorter wait times associated with these systems. Mahmood et al [32] reported a 56.8% reduction in time to see a physician compared with the control group. Dickson et al [31] observed a quicker median time to triage by 15 minutes and a faster time to first identification.

Similarly, Coyle et al [30] reported that their intervention reduced the time to first identification by 4 minutes. However, after adjusting the number of triaged patients, these authors reported no significant change in the time-to-physician measure and time-to-triage [30]. Trivedi et al [34] reported 65% over-triage rates, while Dickson et al [31] reported 59.2%.

## Discussion

### Summary of Main Findings

The review identified 3 primary functions of kiosks in ED settings: self-check-in [30,32,33], self-triage [34], and combined check-in and triage [31]. While digital triage kiosks present a generally safe addition to ED systems, demonstrating low under-triage rates and accurately identifying high-acuity presentations, these findings should be interpreted in the context of a limited evidence base. However, algorithm-generated triage scores often prioritize patient safety and tend to overestimate the urgency of a patient’s condition (over-triage). Consequently, their impact on operational efficacy remains unclear. Although 2 studies reported shorter wait times, the significant potential for over-triage is likely a limiting factor.

### Comparison with Existing Literature

This review reinforces earlier findings that evidence regarding the impact and long-term effectiveness of digital kiosks remains limited. However, it significantly contributes to this sparse evidence base by explicitly examining the impact of 3 different functions and assessing the direct effect of digital self-triage as a tool for decision-making in busy ED environments [22-24].

Kiosks have demonstrated usability, ease of use, and patient satisfaction across various health care settings [22-24]. This work identified studies exclusively from high-income countries, where kiosks are predominantly used in secondary care settings. High-income countries likely support the broader adoption of these technologies due to their advanced health infrastructure and greater resources [22]. Moreover, our results align with previous literature, which states that these technologies tend to over-triage those attending ED [21,35]. Our findings align with a recent independent review of a combined self-check-in and triage tool in a large London NHS emergency department [36]. The review reported an 11-minute reduction in preregistration queue wait times during busy ED periods and a 14% decrease in nurse triage assessment duration. Staff-reported experiences were consistent with the high usability rate noted in our included studies, with 100% agreeing that the tool positively impacted patient flow and safety and 82% feeling that they could perform their roles more effectively with kiosks.

### Strengths and Limitations

This systematic review is the first comprehensive summary of the impact of self-service kiosks on safety and efficacy, highlighting their potential benefits and current limitations. It addresses a critical gap in the literature by offering a detailed narrative synthesis of previously unexplored outcomes.

Despite the extensive search across 3 databases, only 5 articles were eligible for inclusion, leaving significant gaps in understanding triage duration, clinical outcomes, and adverse events. This search identified only 2 studies examining self-triage kiosks, limiting our ability to draw comprehensive conclusions from the data about this function. This is likely due to strict eligibility criteria, as the review focused on testing the practical application of self-triage kiosks in clinical settings rather than the optimization of triage algorithms or simulated environments. Plus, our search retrieved a few studies on self-check-in technologies, further constraining the available evidence. All eligible studies originated from high-income countries, limiting our findings’ generalizability to low- and middle-income settings. Unaccounted variations in terminology and culture may have contributed to this limitation. Additional databases could strengthen the search strategy and identify more relevant literature. Significant variations in kiosk design and functionality complicated outcome comparisons, rendering a meta-analysis unfeasible. The limited number of studies prevented a quantitative subgroup analysis, so outcomes were organized by kiosk function to facilitate a clear comparison. Given the kiosks’ 3 distinct functions, it was determined that comparing only their physical aspects was the most feasible approach. However, this may overlook more detailed analyses, as factors like kiosk design and additional features could offer valuable insights not captured in this review.

Acknowledging the limitations of using nurse-assessed scores as the “gold-standard” comparator for kiosk-generated triage scores is essential. Various factors influence triage decisions, including the nurse’s proficiency, age, experience, and triage training [37]. For example, an experienced nurse may assign a lower acuity score to a patient, relying on their ability to manage high-acuity cases effectively [38]. Clinicians frequently adjust triage scores postassessment, either downgrading or upgrading patients based on further evaluation. This practice further complicates the reliability of nurse-assessed scores as a comparator in these studies and, consequently, in this review. A lack of patient experience or satisfaction information in the included studies represents another limitation.

### Implications for Future Research

Further research is required to expand the limited evidence base concerning digital interventions designed to alleviate ED pressure and support the care of an increasingly complex population of individuals with undifferentiated presentations. To achieve immediate impact, a greater understanding of the safety and efficacy of assessment is necessary to support the ongoing development and implementation of digital triage tools in these settings. Researchers must conduct longitudinal studies to assess the long-term effects of digital triage systems on health outcomes and readmission rates.

Future research should prioritize equity indicators, including age, language, ethnicity, and socioeconomic status, while also considering the diversity of geographical contexts [31]. As low- and middle-income settings require more efficient ED solutions, researchers should investigate digital kiosks in these contexts to better understand their potential impact. As health care systems increasingly implement digital, fast-paced health care settings, it is essential to evaluate digital literacy and its impact on usability. Understanding this relationship is critical to ensuring accessibility for the most vulnerable populations.

This review highlights significant challenges related to over-triage, emphasizing the need for further research on optimizing triage-related decision-making using advanced technologies. The self-triage interventions examined in this review use decision-logic algorithms to triage patients, but researchers should also consider alternatives. Artificial intelligence (AI) capabilities could enable the autostreaming of patients to appropriate care pathways within EDs (eg, same-day emergency care) or to other units or care settings (eg, urgent treatment centers or minor injury units). AI could also facilitate preordering necessary investigations, improving safety, efficiency, and health outcomes. Integrating digital kiosks with self-assessment tools like symptom checkers or chatbots could streamline triage before ED arrival. Further research must explore how this approach could reduce care delays and improve patient flow efficiency. Alternative comparators to nurse-assigned triage scores and composite outcome-based measures independent of this comparator should (eg, intensive care unit admissions) also be explored to ensure accurate real-world evaluation of these technologies. Researchers using nurse-assigned triage scores should incorporate inter-rate reliability checks, blinded reviewers, or consensus scoring to reduce this bias and strengthen the validity of their assessments. In addition, conducting detailed postmarket surveillance of products in clinical use is essential for tracking and analyzing adverse outcomes, ultimately facilitating improvements in system safety and accuracy in digital systems.

### Implications for Policy and Practice

This review indicates that self-service kiosks in EDs can provide safe and effective care, but further development and evaluation are essential to ensure continual improvement. To maximize their impact, particularly in low- and middle-income settings, efforts should focus on strengthening infrastructure and resource availability. In addition, policymakers and regulators must prioritize digital clinical safety and actively discuss the topic to keep pace with the evolving digital landscape and address ED workflow challenges [39,40]. Regulatory bodies must align national guidelines, regulations, and policies with best practices to balance innovation with privacy and security while tackling ethical data concerns. Regulation must keep pace with the dynamic development in AI learning and feedback loops to ensure adequate oversight. Policymakers should incentivize software developers to refine algorithms and minimize triage errors.

Since the COVID-19 pandemic accelerated the adoption of digital solutions, providers have become increasingly receptive to using them, even as digital literacy barriers may still limit accessibility for specific patient groups. Implementing kiosks alongside in-person services could help alleviate ED pressures by allowing staff to quickly identify and prioritize urgent cases, freeing up administrative and reception staff to support patients with complex physical or communication needs. However, integrating kiosks into routine ED service delivery presents inevitable challenges, given the complexity of these healthcare environments. Poor planning, implementation, and user-centered design could increase staff cognitive load and the risk of error [41,42]. Therefore, health care providers must ensure adequate training and system usability for effective implementation to avoid duplication of work and unnecessary costs. Plus, further consideration of the cost-effectiveness of implementing and maintaining these systems in EDs is necessary to ensure their sustainability.

Digital kiosk tools must remain flexible to accommodate diverse ED settings and adapt to local needs, where staff composition and patient populations vary significantly. Providers must engage both patients and staff throughout the integration process to overcome resistance. Health care systems should incorporate real-world feedback mechanisms to support safe, iterative, and human-centered implementation. Finally, with the rapidly evolving technological development, EDs must be prepared for the availability of digital check-in tools with more advanced functions, such as using more advanced algorithms and AI to risk-stratify and stream patients. This review demonstrates that self-triage kiosks already use decision-logic algorithms to support triage, highlighting their role in enhancing patient flow. As these systems evolve, regulators (eg, Medicines and Healthcare Products Regulatory Agency and US Food and Drug Administration) must ensure detailed but flexible assessment of new technologies to support their safe implementation in clinical settings.

### Conclusions

Balancing technological efficacy with personalized care is critical in ensuring positive clinical outcomes and patient well-being. This review demonstrates that while digital self-service kiosks show clear promise, a lack of evidence prevents a definitive assessment of their true impact in EDs. It identifies 3 functions of self-service kiosks: self-check-in, self-triage, and combined self-check-in and triage. Digital triage kiosks likely provide a safe means of accurately identifying and prioritizing patients most in need of care, but their impact on operational efficacy remains unclear. Consequently, researchers must urgently generate more evidence to unlock their potential to alleviate the growing demands on EDs globally. As patient safety becomes integral to digital transformation, health care systems must allocate additional resources to ensure the successful and safe implementation of these tools. As NHS EDs increasingly adopt digital self-check-in triage kiosks, the significant patient throughput offers substantial opportunities for further research and evaluation. Software developers and ED sites should design and plan for structured postimplementation impact assessments to contribute to real-world evidence for these tools.

## Appendix

Multimedia Appendix 1 PRISMA 2020 Checklist for Systematic Reviews.

Multimedia Appendix 2 Search strategies.

Multimedia Appendix 3 Risk of bias assessment for included studies.

## Abbreviations

AGST: algorithm-generated self-triage
AI: artificial intelligence
ED: emergency department
MeSH: Medical Subject Headings
MTS: Manchester Triage Score
NHLBI: National Heart, Lung, and Blood Institute Quality Assessment Tool
NHS: National Health System
PICOS: Population, Intervention, Comparator, Outcome, Study design
PRISMA: Preferred Reporting Items for Systematic Reviews and Meta-Analysis
PROSPERO: International Prospective Register of Systematic Reviews
