# Supplementary material for: Safety and Efficacy of Digital Check-in and Triage Kiosks in Emergency Departments: Systematic Review
Source: J Med Internet Res. 2025 May 21;27:e69528. doi: 10.2196/69528 (PMC12138304; doi:10.2196/69528)
Supplement: Multimedia Appendix 2 [file jmir_v27i1e69528_app2.docx]

**Appendix**

Appendix 1: Search strategies for all databases searched.

**Medline (via OVID)**

| ***Order of search*** | ***Search syntax*** |
| --- | --- |
| ***Emergency Department*** |  |
| 1 | accident and emergency* OR emergency care OR urgent care* |
| 2 | Emergency Medical Services/ OR Emergency Service, Hospital/ |
| 3 | 1 OR 2 |
| ***Kiosks*** | |
| 4 | self-service check-in OR electronic triage OR digital triage OR etriage OR e-triage OR self-triage OR self-assess* OR tele-triage OR (electronic adj2 check-in).tw. |
| 5 | Triage/ OR Diagnostic Self-Evaluation/ OR Self-Assessment/ |
| 6 | 4 OR 5 |
| ***Safety*** | |
| 7 | clinical outcomes OR clinical safety OR misdiagnos* OR error* OR near miss*.tw. |
| 8 | Patient Safety/ OR Patient Harm/ OR Medical Errors/ OR Treatment Outcome/ |
| 9 | Safety Management/ OR Risk Management/ |
| 10 | 7 OR 8 OR 9 |
| ***Efficacy*** | |
| 11 | patient flow OR wait* list* OR wait* time* OR triage time OR efficiency OR triage efficiency.tw. |
| 12 | Efficacy, Efficiency, Organisational/ OR Efficiency/ OR Workflow/ OR Workload/ |
| 13 | Waiting Lists/ OR Time-to-Treatment/ |
| 14 | 11 OR 12 OR 13 |
|  | |
| 15 | 3 AND 6 AND 10 AND 14 |

**Embase (via OVID)**

| ***Order of search*** | ***Search syntax*** |
| --- | --- |
| ***Emergency Department*** | |
| 1 | accident and emergency* OR emergency care OR urgent care*.tw. |
| 2 | emergency health service/ OR hospital emergency service/ |
| 3 | 1 OR 2 |
| ***Kiosk*** | |
| 4 | self-service check-in OR electronic triage OR digital triage OR etriage OR e-triage OR self-triage OR self-assess* OR tele-triage OR (electronic adj2 check-in).tw. |
| 5 | triage/ OR diagnostic self evaluation/ OR self assessment/ |
| 6 | 4 OR 5 |
| ***Safety*** | |
| 7 | clinical outcomes OR clinical safety OR misdiagnos* OR error* OR near miss*.tw. |
| 8 | patient safety/ OR patient harm/ OR medical error/ OR treatment outcome/ |
| 9 | safety management/ OR risk management/ |
| 10 | 7 OR 8 OR 9 |
| ***Efficacy*** | |
| 11 | patient flow OR wait* list* OR wait* time* OR triage time OR efficiency OR triage efficiency.tw. |
| 12 | organizational efficiency/ OR workflow/ OR workload/ |
| 13 | waiting list/ OR time to treatment/ |
| 14 | 11 OR 12 OR 13 |
|  | |
| 15 | 3 AND 6 AND 10 AND 14 |

**Web of Science**

| ***Order of search*** | ***Search syntax*** |
| --- | --- |
| ***Emergency Department*** | |
| 1 | TS=("accident and emergency*" OR "emergency care" OR "urgent care*") |
| ***Kiosk*** | |
| 2 | TS=("self-service check-in" OR "electronic triage" OR "digital triage" OR "etriage" OR "e-triage" OR "self-triage" OR "self-assess*" OR "tele-triage" OR ("electronic" NEAR/2 "check-in")) |
| ***Safety*** | |
| 3 | TS=("clinical outcomes" OR "clinical safety" OR "misdiagnos*" OR "error*" OR "near miss*") |
| 4 | TS=("patient safety" OR "patient harm" OR "medical errors" OR "treatment outcome" OR "safety management" OR "risk management") |
| 5 | 3 OR 4 |
| ***Efficacy*** | |
| 6 | TS=("patient flow" OR "wait* list*" OR "wait* time*" OR "triage time" OR "efficiency" OR "triage efficiency" OR "workflow" OR "workload" OR "time-to-treatment") |
|  | |
| 7 | 1 AND 2 AND 5 AND 6 |
