# Supplementary material for: Safety and Efficacy of Digital Check-in and Triage Kiosks in Emergency Departments: Systematic Review
Source: J Med Internet Res. 2025 May 21;27:e69528. doi: 10.2196/69528 (PMC12138304; doi:10.2196/69528)
Supplement: Multimedia Appendix 3 [file jmir_v27i1e69528_app3.docx]

**Table S1.** Quality assessment of the included studies.

|  | \| Author \| Study design \| Q1 \| Q2 \| Q3 \| Q4 \| Q5 \| Q6 \| Q7 \| Q8 \| Q9 \| Q10 \| Q11 \| Q12 \| Q13 \| Q14 \| \| --- \| --- \| --- \| --- \| --- \| --- \| --- \| --- \| --- \| --- \| --- \| --- \| --- \| --- \| --- \| --- \| \| Coyle et al, 2019 [1] \| Prospective randomized controlled trial design \| **✓** \| N/A \| N/A \| **X** \| **X** \| **✓** \| **X** \| N/A \| **✓** \| N/A \| **✓** \| N/A \| N/A \| N/A \| \| Dickson et al, 2022 [2] \| Retrospective, observational design \| **✓** \| **✓** \| **✓** \| **✓** \| **X** \| **✓** \| **✓** \| **✓** \| **✓** \| **X** \| **✓** \| N/A \| N/A \| **X** \| \| Mahmood et al, 2020 [3] \| Cross-sectional, retrospective, observational design \| **✓** \| **✓** \| **X** \| **✓** \| **X** \| **✓** \| **✓** \| **X** \| **✓** \| **X** \| **✓** \| N/A \| N/A \| **✓** \| \| Tabriz et al, 2020 [4] \| Retrospective analysis, observational \| **✓** \| **✓** \| **X** \| **✓** \| **X** \| **✓** \| **✓** \| **✓** \| **✓** \| **X** \| **✓** \| N/A \| N/A \| **✓** \| \| Trivedi et al, 2021 [5] \| Prospective, pilot design \| **✓** \| **✓** \| **✓** \| **✓** \| **X** \| **✓** \| **✓** \| **X** \| **✓** \| **X** \| **✓** \| N/A \| **✓** \| **X** \| |
| --- | --- | --- | --- | --- | --- | --- | --- | --- | --- | --- | --- | --- | --- | --- | --- | --- | --- | --- | --- | --- | --- | --- | --- | --- | --- | --- | --- | --- | --- | --- | --- | --- | --- | --- | --- | --- | --- | --- | --- | --- | --- | --- | --- | --- | --- | --- | --- | --- | --- | --- | --- | --- | --- | --- | --- | --- | --- | --- | --- | --- | --- | --- | --- | --- | --- | --- | --- | --- | --- | --- | --- | --- | --- | --- | --- | --- | --- | --- | --- | --- | --- | --- | --- | --- | --- | --- | --- | --- | --- | --- | --- | --- | --- | --- | --- | --- | --- |

**Questions for the Tool for Quality Assessment (National Heart, Lung and Blood Institute Quality Assessment Tool)**

Observational Cohort and Cross-Sectional Studies

1. Was the research question or objective in this paper clearly stated?

2. Was the study population clearly speciﬁed and deﬁned?

3. Was the participation rate of eligible persons at least 50%?

4. Were all the subjects selected or recruited from the same or similar populations (including the same time)? Were inclusion and exclusion criteria for being in the study prespeciﬁed and applied uniformly to all participants?

5. Was a sample size justiﬁcation, power description, or variance and eﬀect estimates provided?

6. For the analyses in this paper, were the exposure(s) of interest measured prior to the outcome(s) being measured?

7. Was the timeframe suﬃcient so that one could reasonably expect to see an association between exposure and outcome if it existed?

8. For exposures that can vary in amount or level, did the study examine diﬀerent levels of the exposure as related to the outcome (e.g., categories of exposure, or exposure measured as continuous variable)?

9. Were the exposure measures (independent variables) clearly deﬁned, valid, reliable, and implemented consistently across all study participants?

10. Was the exposure(s) assessed more than once over time?

11. Were the outcome measures (dependent variables) clearly deﬁned, valid, reliable, and implemented consistently across all study participants?

12. Were the outcome assessors blinded to the exposure status of participants?

13. Was loss to follow-up amer baseline 20% or less?

14. Were key potential confounding variables measured and adjusted statistically for their impact on the relationship between exposure(s) and outcome(s)?

Controlled Intervention Studies

1. Was the study described as randomized, a randomized trial, a randomized clinical trial, or an RCT?

2. Was the method of randomization adequate (i.e., use of randomly generated assignment)?

3. Was the treatment allocation concealed (so that assignments could not be predicted)?

4. Were study participants and providers blinded to treatment group assignment?

5. Were the people assessing the outcomes blinded to the participants' group assignments?

6. Were the groups similar at baseline on important characteristics that could aﬀect outcomes (e.g., demographics, risk factors, co-morbid conditions)?

7. Was the overall drop-out rate from the study at endpoint 20% or lower of the number allocated to treatment?

8. Was the diﬀerential drop-out rate (between treatment groups) at endpoint 15 percentage points or lower?

9. Was there high adherence to the intervention protocols for each treatment group?

10. Were other interventions avoided or similar in the groups (e.g., similar background treatments)?

11. Were outcomes assessed using valid and reliable measures, implemented consistently across all study participants?

12. Did the authors report that the sample size was suﬃciently large to be able to detect a diﬀerence in the main outcome between groups with at least 80% power?

13. Were outcomes reported or subgroups analyzed prespeciﬁed (i.e., identiﬁed before analyses were conducted)?

14. Were all randomized participants analyzed in the group to which they were originally assigned, i.e., did they use an intention-to-treat analysis?

### **References**

1. Coyle N, Kennedy A, Schull MJ, Kiss A, Hefferon D, Sinclair P, Alsharafi Z. The use of a self-check-in kiosk for early patient identification and queuing in the emergency department. CJEM 2019 Nov; 21(6):789-792.
2. Dickson SJ, Dewar C, Richardson A, Hunter A, Searle S, Hodgson LE. Agreement and validity of electronic patient self-triage (eTriage) with nurse triage in two UK emergency departments: a retrospective study. Eur J Emerg Med 2022; 29(1):49-55.
3. Mahmood A, Wyant DK, Kedia S, Ahn SN, Powell MP, Jiang Y, Bhuyan SS. Self-check-in kiosks utilization and their association with wait times in emergency departments in the United States. J Emerg Med 2020; 58(5):829-840.
4. Alishahi Tabriz A, Trogdon JG, Fried BJ. Association between adopting emergency department crowding interventions and emergency departments' core performance measures. Am J Emerg Med 2020; 38(2):258-265.
5. Trivedi S, Littmann J, Stempien J, Kapur P, Bryce R, Betz M. A comparison between computer-assisted self-triage by patients and triage performed by nurses in the emergency department. Cureus 2021; 13(3):e14002.
